# New Molecular Insights of Insulin in Diabetic Cardiomyopathy

**DOI:** 10.3389/fphys.2016.00125

**Published:** 2016-04-12

**Authors:** Francisco Westermeier, Jaime A. Riquelme, Mario Pavez, Valeria Garrido, Ariel Díaz, Hugo E. Verdejo, Pablo F. Castro, Lorena García, Sergio Lavandero

**Affiliations:** ^1^Faculty of Chemical and Pharmaceutical Sciences and Faculty of Medicine, Advanced Center for Chronic Diseases, University of ChileSantiago, Chile; ^2^Faculty of Medicine, Advanced Center for Chronic Diseases, Pontifical Catholic University of ChileSantiago, Chile; ^3^Division of Cardiovascular Diseases, Faculty of Medicine, Pontifical Catholic University of ChileSantiago, Chile; ^4^Department of Internal Medicine (Division of Cardiology), University of Texas Southwestern Medical CenterDallas, TX, USA

**Keywords:** insulin, diabetic cardiomyopathy, miRNAs, exosomes, adenosine, adenosine receptors

## Abstract

Type 2 diabetes mellitus (T2DM) is a highly prevalent disease worldwide. Cardiovascular disorders generated as a consequence of T2DM are a major cause of death related to this disease. Diabetic cardiomyopathy (DCM) is characterized by the morphological, functional and metabolic changes in the heart produced as a complication of T2DM. This cardiac disorder is characterized by constant high blood glucose and lipids levels which eventually generate oxidative stress, defective calcium handling, altered mitochondrial function, inflammation and fibrosis. In this context, insulin is of paramount importance for cardiac contractility, growth and metabolism and therefore, an impaired insulin signaling plays a critical role in the DCM development. However, the exact pathophysiological mechanisms leading to DCM are still a matter of study. Despite the numerous questions raised in the study of DCM, there have also been important findings, such as the role of micro-RNAs (miRNAs), which can not only have the potential of being important biomarkers, but also therapeutic targets. Furthermore, exosomes also arise as an interesting variable to consider, since they represent an important inter-cellular communication mechanism and therefore, they may explain many aspects of the pathophysiology of DCM and their study may lead to the development of therapeutic agents capable of improving insulin signaling. In addition, adenosine and adenosine receptors (ARs) may also play an important role in DCM. Moreover, the possible cross-talk between insulin and ARs may provide new strategies to reverse its defective signaling in the diabetic heart. This review focuses on DCM, the role of insulin in this pathology and the discussion of new molecular insights which may help to understand its underlying mechanisms and generate possible new therapeutic strategies.

## Diabetic cardiomyopathy: Pathophysiology and clinical features

Over the past two decades, the prevalence of type 2 diabetes mellitus (T2DM) has steadily increased as a consequence of higher rates of obesity, an increase in sedentary lifestyle, and changes in the environment. If unaddressed, nearly half a billion people will be burdened by this chronic disease in 2030 (Shaw et al., 2010). At one time, whether T2DM contributed to cardiac dysfunction in diabetic patients was a matter of debate (Rubler et al., 1972). However, diabetic cardiomyopathy (DCM)—with a prevalence of 12% in T2DM patients (Bertoni et al., 2004)—is now recognized as a serious complication of T2DM resulting from sustained hyperglycaemia and hyperlipidemia that leads to increases in cardiac oxidative stress, inflammation, and myocardial fibrosis, as well as detrimental changes in Ca^2+^ handling and mitochondrial function (Bugger and Abel, 2014; Jia et al., 2016). Several signaling pathways converge in the development of DCM, and the goal of finding suitable therapeutic targets remains a challenge. From a clinical standpoint, DCM is defined as the presence of left ventricular (LV) dysfunction in patients with T2DM in the absence of arterial hypertension, coronary artery disease (CAD) or evidence of any other structural cardiac disease (Battiprolu et al., 2010, 2013; Maisch et al., 2011; Adeghate and Singh, 2014; Isfort et al., 2014). Its clinical presentation ranges from early-stage DCM, which is characterized by abnormal myocardial energy metabolism, diastolic dysfunction and reduced LV strain to overt heart failure (HF), associated with cardiomyocyte hypertrophy, myocardial fibrosis and cardiomyocyte death (Voulgari et al., 2010; Falcao-Pires and Leite-Moreira, 2012; Battiprolu et al., 2013; Dhalla et al., 2014; Go et al., 2014; Isfort et al., 2014).

The first stage of DCM is clinically asymptomatic, but is evidenced by a characteristic diastolic dysfunction (increased ventricular stiffness, left atrial enlargement, and elevated LV end-diastolic pressure) (Battiprolu et al., 2010). The prevalence of diastolic dysfunction is several times higher in T2DM patients than in sex- and age-matched populations and can impact up to 40% of T2DM patients (Poulsen et al., 2010). Several mechanisms contribute to ventricular stiffness in T2DM: for instance, sustained hyperglycemia promotes formation of advanced glycation end products (AGEs), which are particularly prone to cross-linking with extracellular proteins, such as collagen and elastin, altering their structural properties and impairing cardiac relaxation (Murarka and Movahed, 2010; Jia et al., 2016).

The second stage of DCM is characterized by cardiac remodeling, LV hypertrophy (LVH), and the emergence of clinical indications of HF (Ozasa et al., 2008); however, whether LV systolic dysfunction ultimately develops is a matter of debate. Although some authors have suggested that there is an association between systolic dysfunction and long-standing diabetes (Mbanya et al., 2001), most publications indicate that resting systolic function is normal in the majority of T2DM patients, as assessed by LV ejection fraction (LVEF), although some subjects may exhibit systolic impairment during exercise (Vered et al., 1984). However, LVEF can be a poor approximation of cardiac contractility and may not detect minor changes in myocardial function (Sonoyama et al., 2007). These abnormalities in myocardial function correlate closely with changes in Ca^2+^ handling, caused in part by AGEs that decrease mechanical efficiency of the sarcomere and impair electromechanical coupling, ultimately leading to reduced contractility (Bugger and Abel, 2014). In addition, the activity of the sarco(endo)plasmic reticulum Ca^2+^-ATPase isoform 2 (SERCA2), the ryanodine receptor (RyR) and Na^+^/Ca^2+^ exchanger (NCX1) are markedly reduced in DCM, leading to diminished release of Ca^2+^ from endoplasmic reticulum stores and slowed replenishment of these stores during relaxation. These changes, in turn, lead to elevated end-diastolic Ca^2+^ levels and a progressive decrease in the frequency of Ca^2+^ transients (Zhao et al., 2014). There is evidence that hyperglycemia may activate caspase-3 directly (Cai et al., 2002) causing apoptosis of cardiomyocytes and increased interstitial fibrosis (Fiordaliso et al., 2000). Thus, increased cardiomyocyte apoptosis may be a critical step in the transition from compensated to decompensated HF (Frustaci et al., 2000). It has also been proposed that insulin-resistance in DCM may be related to increased risk of cardiac LVH or HF (Abel et al., 2012). Altered myocardial insulin signaling is a hallmark of DCM (Battiprolu et al., 2012; Bugger and Abel, 2014; Jia et al., 2016); however, the molecular mechanisms that have been proposed to contribute to the development of insulin resistance in DCM are not fully understood. In this review, we summarize the current knowledge about cardiac insulin signaling, with emphasis on new molecular insights regarding its role in DCM.

## Insulin signaling in the normal heart

An appreciation for the role of insulin in glucose homeostasis began with its discovery in 1922 (Banting et al., 1922). The ability of insulin to induce glucose uptake (Levine et al., 1949) by activating specific cell surface receptors on target tissues (Kahn et al., 1974; Ebina et al., 1985; Ullrich et al., 1985), thus, triggering the translocation of the glucose transporter 4 (GLUT4) to the plasma membrane (Cushman and Wardzala, 1980; Suzuki and Kono, 1980), is well documented. However, much remains to be learned regarding both the physiological and pathophysiological impact of insulin signaling in the diversity of tissues, such as adipose tissue and skeletal muscle, upon which it acts (Leto and Saltiel, 2012). Although a comprehensive understanding of the mechanisms underlying insulin action in the heart is only just emerging, there is abundant evidence that insulin impacts cardiac metabolism by influencing a wide range of cellular processes, such as glucose transport, glycolysis, glycogen synthesis (Abel, 2004), cardiac hypertrophy, protein synthesis, lipid metabolism (Belke et al., 2002). Moreover, insulin also participates in myocardial contractility (Fu et al., 2014), protection against ischemia-induced necrotic death (Diaz et al., 2015), autophagy (Riehle et al., 2013), and cell survival, either directly or through the closely associated insulin-like growth factor-1 (IGF-1) (Troncoso et al., 2014).

### Cardiac metabolism

**Figure 1:**
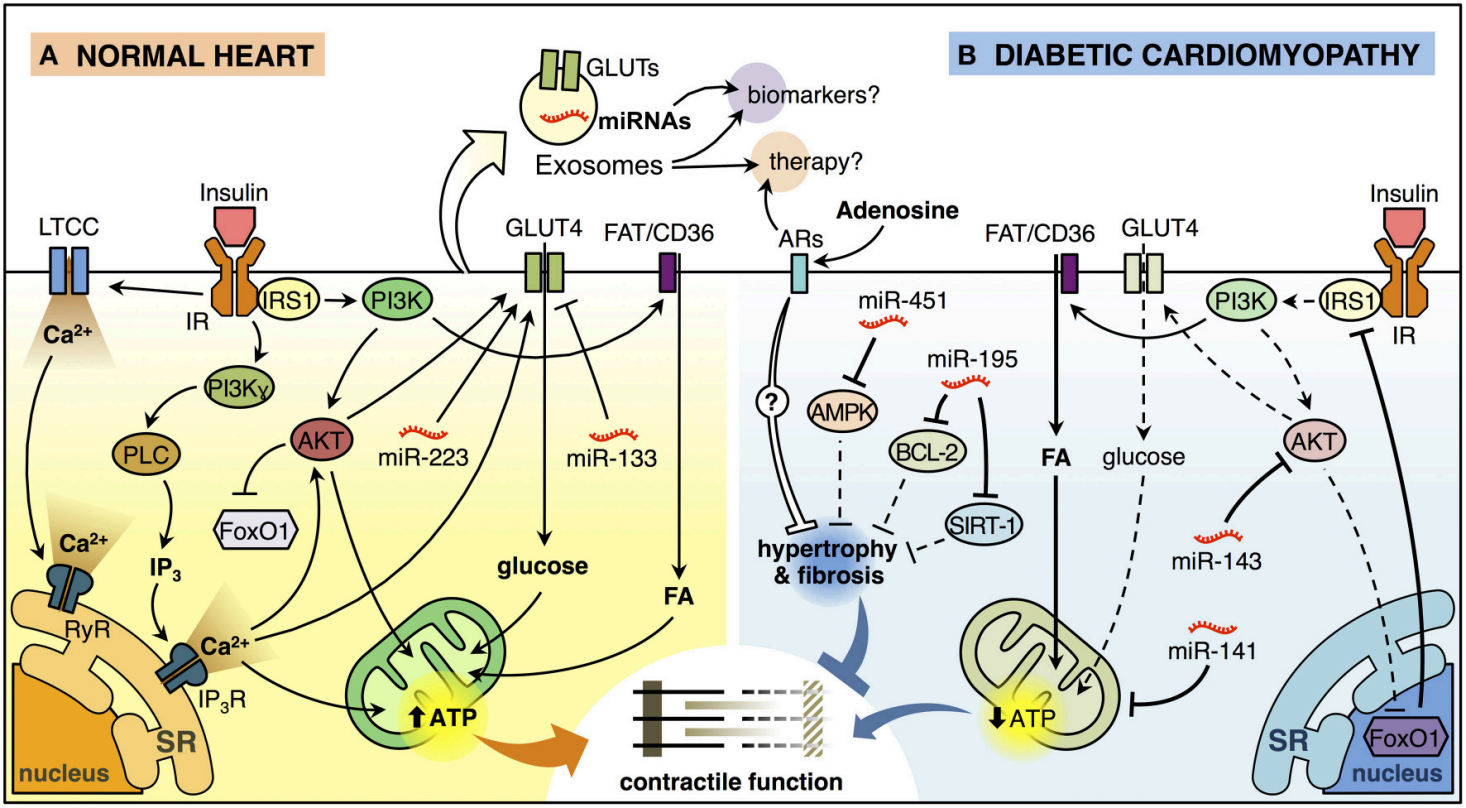
**Insulin signaling in the normal heart and diabetic cardiomyopathy**. **(A)** Insulin binding to its receptor (insulin receptor, IR) promotes a biphasic [Ca^2+^]_i_ response in cardiomyocytes. The first phase of insulin-dependent [Ca^2+^]_i_ increase involves extracellular Ca^2+^ influx through L-type calcium channel (LTCC) and activation of ryanodine receptor (RyR) Ca^2+^ channel which in turn releases Ca^2+^ from the sarcoendoplasmic reticulum (SR). The second phase involves a non-inhibitory G protein coupled receptor (Gβγ subunits) that activates downstream effectors, including phosphatidylinositol 3-kinase (PI3K)γ and phospholipase C (PLC). PLC generates inositol-1,4,5-trisphosphate (IP_3_) that opens the IP_3_-ligated Ca^2+^channels in the SR. These mechanisms trigger the translocation of glucose transporter 4 (GLUT4) from an intracellular store to the plasma membrane (PM) and increase glucose uptake. Insulin also induces the translocation of the fatty acid (FA) transporter FAT/CD36 to the PM trough PI3K activation promoting the FA uptake. Increased lipid and glucose uptake increase mitochondrial oxidative metabolism generating adenosine triphosphate (ATP) that supports myocardial contractile function. Insulin activation of AKT, downstream of PI3K, inactivates the transcription factor FoxO1. In addition, miR-223 stimulates and miR-133 reduces GLUT4-dependent glucose uptake. **(B)** Insulin signaling is deficient in diabetic cardiomyopathy (DCM). FoxO1-dependent downregulation of insulin receptor substrate 1 (IRS1) and AKT are associated with reduced insulin-induced GLUT4 translocation to the PM and lower glucose uptake. At the same time, cardiomyocytes accumulate lipids, reducing mitochondrial oxidative metabolism and promoting mitochondrial uncoupling, which in turn, affects cardiomyocyte function. Also, miR-143 inhibits insulin signaling, whereas miR-141 may impact mitochondrial function and the production of ATP. On the other hand, miR-451 is associated with inhibition of AMPK, while miR-195 inhibits the expression of BCL-2 and SIRT-1. Both of these miRNAs are associated with hypertrophy, which will consequently generate contractile dysfunction. Adenosine may have a paracrine effect on cardiomyocytes, which could reduce hypertrophy and myocardial fibrosis through the activation of adenosine receptors (ARs). Exosomes can ferry miRNAs and proteins such as GLUT4 from one cell to another and therefore, they can be used as potential biomarkers or therapeutic agents/targets for DCM.

Insulin-stimulated glucose uptake in cardiomyocytes is mediated primarily through mobilization of glucose transporter 4 (GLUT4). Basal cardiac glucose uptake is mediated by glucose transporter 1 (GLUT1), however, contraction-mediated activation of GLUT4 translocation may also contribute significantly to myocardial glucose uptake (Abel, 2004). We have reported that GLUT4-mediated glucose uptake in cardiomyocytes can be activated through a G protein, phosphatidylinositol 3-kinase (PI3K)γ and 1,4,5-inositol-triphosphate (IP_3_) receptor (IP_3_R) signaling axis, leading to generation of a cytoplasmic Ca^+2^ signal that mobilizes GLUT4 (Contreras-Ferrat et al., 2010; Figure 1A). Although the mechanisms of insulin regulation of cardiac GLUT4 are not yet fully defined, recent data show that constitutive activation of PI3K and AKT can reduce glucose uptake in mouse cardiomyocytes through a mechanism that does not involve GLUT4 translocation, suggesting that insulin impacts GLUT4 activity beyond the ability to regulate translocation to the plasma membrane (Zhu et al., 2013a). Moreover, in the heart of mice with a cardiomyocyte-selective deletion of GLUT4 (G4H^−∕−^), there was a reduction in the levels of insulin-responsive aminopeptidase (IRAP) which was related to impaired GLUT4 endosomal trafficking (Abel et al., 2004), further suggesting that GLUT4 may play a role in regulating glycolysis beyond its ability to transport glucose (Tian and Abel, 2001), evidence that highlight the critical role of GLUT4 and GLUT1 in the insulin-mediated glucose transport on cardiac metabolism.

### Cardiac contractility

Several animal studies have shown the cardioprotective effects of insulin (Das, 2003). In addition, clinical studies have suggested an association between insulin resistance and HF (Arnlov et al., 2001; Ingelsson et al., 2005). However, excessive cardiac insulin signaling can exacerbate systolic dysfunction induced by pressure overload in mice (Shimizu et al., 2010). Cardiac specific insulin receptor knockout (CIRKO) hearts show a mild reduction in cardiac function (Belke et al., 2002) and developed more severe contractile dysfunction compared with control hearts in response to isoproterenol exposure, a combined β_1_- and β_2_-adrenergic receptor (βAR) agonist (McQueen et al., 2005). Fu et al. proposed an interesting model for crosstalk between the insulin receptor (IR) and β_2_AR signaling in the heart (complex IR/β_2_AR) that could impact cardiac contractility (Fu et al., 2014). In their model, increased IR signaling promotes dissociation of the IR/β_2_AR complex, leading to an impaired contractile response to β_2_AR subsequent activation. This effect was seen in isolated neonatal and adult cardiomyocytes, as well as Langendorff-perfused hearts (Fu et al., 2014). Moreover, CIRKO mice, which are deficient for IR signaling, display defective electrical impulse propagation, elevated K^+^ extracellular concentrations (Punske et al., 2004), and diminished levels of several key K^+^ channel components involved in ventricular repolarization (Lopez-Izquierdo et al., 2014). Moreover, insulin can diminish markers of cytosolic oxidative stress (Li et al., 2012) and improve impaired cardiac electrical activity during hypoxia in G4H^−∕−^ hearts (Sohn et al., 2013). Altogether, this evidence suggests a link between insulin signaling and the electrical function in the heart independent of its activation of GLUT4-dependent glucose uptake.

### Cardiac growth

In the adult heart, myocardial growth is achieved primarily through hypertrophic expansion of individual cardiomyocytes (Pasumarthi and Field, 2002). Heart size increases during post-natal development in response to several stimuli (Olson and Schneider, 2003), and these responses can be classified either as “pathological” or “physiological” (Richey and Brown, 1998). Insulin is considered an anabolic hormone that promotes protein synthesis, cell growth and hypertrophy (Shiojima et al., 2002). CIRKO mice are characterized by a 30% reduction in heart size (Belke et al., 2002), consistent with the effects observed in cardiomyocytes with reduced PI3K/AKT signaling, where insulin-stimulated hypertrophy is blunted (Shioi et al., 2000; Shiojima et al., 2002). G4H^−∕−^ mice developed modest cardiac hypertrophy, with preserved contractile function, that is associated with an increase in cardiomyocyte size (Abel et al., 1999). Another study conducted by O'Neill et al. suggests that PI3K activates AKT-independent signaling pathways that mediate metabolic and mitochondrial adaptations during physiological cardiac hypertrophy (O'Neill et al., 2007). Cardiomyocyte-specific overexpression of constitutively activated mechanistic target of rapamycin (mTOR), an important target and modulator of the insulin-stimulated AKT pathway, did not elicit a significant increase in adult heart weight (Shen et al., 2008), even though mTOR is essential for cardiac development and growth during embryogenesis (Zhu et al., 2013b). Cardiomyocytes treated with the peroxisome proliferator-activated receptor (PPAR)γ agonist troglitazone also respond by increasing protein content (Bell and Mcdermott, 2000); however, the hypertrophic response of cardiomyocytes to PPARγ agonists does not appear to be mediated by changes in myocardial insulin signaling (Sena et al., 2007).

Studies of mice with cardiomyocyte-specific deletion of either insulin receptor substrate 1 (IRS1) (CIRS1KO mice) or IRS2 (CIRS2KO mice) reported that at baseline, adult CIRS1KO hearts were reduced in size, whereas CIRS2KO hearts exhibited cardiac hypertrophy. However, following a swim training exercise regimen, mitochondrial capacity and substrate oxidation rates increased in wild-type controls, but not in IRS-deficient hearts, suggesting that although different IRS isoforms may perform different roles during resting conditions, both are required for the hypertrophic and metabolic adaptation to exercise (Riehle et al., 2014). In summary, these studies suggest an important role for insulin signaling in physiological cardiac growth, however studies by Ikeda et al. indicated that in response to exercise IGF-1 may actually activate both the IGF-1 receptor (IGF-1R) and IR in the heart (Ikeda et al., 2009). Thus, deciphering the crosstalk between the IGF-1 and insulin signaling pathways will be crucial to understanding the role of IR in exercise-induced cardiac hypertrophy.

## Insulin signaling in diabetic cardiomyopathy

Defective insulin signaling in DCM has been described in both animal models (Wright et al., 2009) and humans (Cook et al., 2010), primarily observed as reduced GLUT4 translocation to the plasma membrane. Cardiac dysfunction induced by pressure overload in mice is associated with insulin resistance, hyperinsulinemia and increased AKT activation (Shimizu et al., 2010). Hearts from obese and insulin-resistant rodent models (*ob/ob* and *db/db* mice) exhibit higher rates of fatty acid (FA) oxidation and MVO_2,_ but lower rates of glucose oxidation and cardiac efficiency, which precede the development of hyperglycemia (Mazumder et al., 2004; Buchanan et al., 2005). Moreover, CIRKO mice exhibit decreased LV function associated with mitochondrial dysfunction and reduced mitochondrial FA oxidative capacity following a myocardial infarct (Sena et al., 2009), as well as an enhanced decline in cardiac function when treated with streptozotocin (STZ) compared to wild-type mice (Bugger et al., 2012). In addition, these animals have defects in FA and pyruvate metabolism and reduced tricarboxylic acid flux associated with mitochondrial uncoupling. Thus, altered insulin signaling in the heart may contribute directly to mitochondrial dysfunction in the setting of obesity and T2DM (Boudina et al., 2009). In contrast, the type 1 diabetic Akita mouse model (Akita) is characterized by normal cardiac function and preserved myocardial insulin sensitivity, and does not exhibit FA-induced mitochondrial uncoupling (Bugger et al., 2008). These results suggest that the activity of pathways controlling cardiac mitochondrial function may be different in insulin-responsive type 1 and insulin-resistant T2DM hearts.

Interestingly, FoxO (forkhead box-containing protein, O subfamily) proteins have been described as important regulators of several cellular processes in the myocardium such as cardiac growth (Skurk et al., 2005; Ni et al., 2006), and autophagy (Sengupta et al., 2009; Schips et al., 2011). In addition, Ni et al. (2007) showed that FoxO activation leads to altered insulin signaling and decreased glucose uptake in cardiac myocytes (Ni et al., 2007). In this sense, activation of FoxO1 in response to the HFD was directly related to down-regulation of IRS1 activity, decreased AKT signaling, and insulin resistance, suggesting that FoxO1 might be a key step in the development of DCM preventing cardiac dysfunction by restoring insulin responsiveness (Battiprolu et al., 2012; Figure 1B). Recently, it has been shown that impaired cardiac insulin signaling FoxO1-mediated stimulates the β-myosin heavy chain (β-MHC) expression promoting cardiac dysfunction (Qi et al., 2015). Thus, FoxO1 plays a key role in the control of cardiac insulin signaling both in physiological and pathophysiological conditions, and it might prove a novel therapeutic or preventive strategy for treating individuals with DCM.

On the other hand, FAs are the major substrate for ATP synthesis in the heart; nevertheless, excess lipid accumulation is associated with decreased cardiac function in obesity and diabetes (Finck et al., 2002, 2003). In this context, insulin is also able to induce FA uptake in cardiomyocytes, promoting the translocation of the FA transporter FAT/CD36 (Glatz et al., 2006; Coort et al., 2007) to the plasma membrane mediated by PI3K activation (Luiken et al., 2002; Figure 1A). Overexpression of PPARγ led to cardiac lipid accumulation and cardiomyopathy associated with increased FAT/CD36 mRNA, but this effect was antagonized by WY-14,643, a potent PPARα agonist (Son et al., 2010). Nevertheless, comprehensive understanding of the relationship between PPARs and insulin in DCM are not yet fully understood (Jia et al., 2016). The heart can metabolize either non-esterified FA (bound to albumin) or FA obtained from lipolysis of lipoprotein-bound triglycerides (TG). Hearts from mice with cardiomyocyte-specific knockout of lipoprotein lipase (LpL) showed reduced FA oxidation and increased glucose oxidation, associated with increased expression of GLUT4, and reduced expression of pyruvate dehydrogenase kinase 4 (PDK4), suggesting that heart is able to adapt by balancing between the use of glucose and FA (Augustus et al., 2006). However, recent studies indicate that deletion of mTOR impairs myocardial palmitate oxidation and reduces expression of several genes associated with FA metabolism while also reducing insulin-stimulated AKT Ser^473^ phosphorylation (Zhu et al., 2013c). Thus, intact mTOR signaling may be essential for maintaining appropriate control over substrate usage. In addition, cardiomyocytes from HFD-fed mice exhibit increased FA oxidation rates and myocardial oxygen consumption (MVO_2_), correlating with reduced GLUT4 content and impaired GLUT4 translocation in response to insulin (Wright et al., 2009; Figure 1B) and consistent with earlier studies involving the response of cardiomyocytes to the adipocytokine resistin (Graveleau et al., 2005). In summary, cardiac lipotoxicity involves detrimental effects that may adversely impact cardiomyocyte function and participate in progression of DCM.

### New insights

#### miRNAs

miRNAs are small, single-strand non-coding RNA, that are transcribed by RNA polymerase II (Pol II), generating a primary miRNA (pri-miRNA). In the nucleus, the RNase III endonuclease Drosha and the double-stranded RNA-binding domain protein DGCR8/Pasha cleave the pri-miRNA to produce the precursor miRNA (pre-miRNA) of ~70-nt. Exportin-5 transports the pre-miRNA into the cytoplasm, where it is cleaved by another RNase III endonuclease, Dicer, together with the protein TRBP/Loquacious, releasing miRNA containing a 21- to 23-nt (Bushati and Cohen, 2007; Pasquinelli, 2012). They serve as important post-transcriptional regulators that down-regulate the expression of their target genes (Pritchard et al., 2012) The exact and detailed role of miRNAs in the heart is far from being completely understood, but there is mounting evidence showing that alterations in many miRNAs correlate with the development and progression of human diseases, including cardiovascular diseases, such as DCM (Asrih and Steffens, 2013). In this context, Zheng et al. (2015) found that miR-195 is up-regulated in the hearts of C57BL/6 mice treated with STZ and this was associated with a decrease expression of its target proteins B cell leukemia/lymphoma 2 (BCL-2) and sirtuin 1 (SIRT-1) (Zheng et al., 2015). Moreover, inhibition of miR-195 inhibited apoptosis, reduced oxidative stress and hypertrophy in diabetic hearts. Additionally, cardiac function and coronary blood flow was improved and the expression of BCL-2 and SIRT-1 was increased, suggesting that targeting miR-195 may attenuate DCM (Zheng et al., 2015). In order to further elaborate on the subject, we briefly discuss about a few miRNAs and their potential role in DCM. The models used, beneficial or detrimental effect on cardiac function and possible mechanisms suggested so far are summarized in Table 1.

**Table 1:** **Effects of microRNAs (miRs) on diabetic cardiomyopathy (DCM) models: beneficial or detrimental effect on cardiac function and possible mechanisms**.

**miR**	**Model**	**Cardioprotective effect**	**Proposed mechanism**	**References**
195	STZ mice	↓	↑ Apoptosis ↑ Oxidative stress ↑ Hypertrophy ↓ Coronary blood flow	Zheng et al., 2015
133a,b	NRCs	↓	↓ Glucose uptake	Horie et al., 2009
133a	STZ mice	↑	↓ TGF-β1 ↓ SMAD2 ↓ p-ERK1/2	Chen et al., 2014
223	NRCs	↑	↑ Glucose uptake	Lu et al., 2010
141	HL-1, STZ mice	↓	↓ ATP production	Baseler et al., 2012
451	HFD-fed mice, NRCs	↓	↓ LKB1/AMPK	Kuwabara et al., 2015
143	HL-1, ARCs	↓	↓ p-AKT ↓ ORP8 ↓ Glucose uptake	Blumensatt et al., 2013
29a,b,c	HL-1, ZDF rat	↓	↓ mTORC1/MCL-1	Arnold et al., 2014
322	*ob/ob* mice	↑	↓ p-AKT	Marchand et al., 2016

*Abbreviations: AKT, protein kinase B; AMPK, AMP-activated protein kinase; ARCs, adult rat cardiomyocytes; ERK1/2, extracellular signal-regulated protein kinases 1 and 2; LKB1, liver kinase B1; MCL-1, myeloid leukemia cell differentiation protein; mTORC1, mammalian target of rapamycin complex 1; NRCs, neonatal rat cardiomyocytes; ORP8, Oxysterol-binding protein-related protein 8; SMAD2, SMAD family member 1; STZ, streptozotocin; TGF-b1, transforming growth factor beta 1; ZDF, Zucker Diabetic Fatty rat. ↓, Decreased; ↑, Increased*.

miR-133a,b expression have been shown to participate in metabolic control by reducing the expression of GLUT4 and insulin-mediated glucose uptake in neonatal rat cardiomyocytes (NRCs) (Horie et al., 2009). In contrast, Chen et al. (2014) found that miR-133a expression was reduced in the hearts of diabetic mice and this was associated with an increase in the expression of fibrosis markers. Moreover, they observed that overexpression of miR-133a in the heart attenuated cardiac fibrosis, which is one of the central causes for the HF generated by DCM (Chen et al., 2014). Considering the apparent contradiction between these two studies performed in different models, the results should be analyzed with precaution and further *in vivo* studies are required to fully clarify the role of miR-133a in DCM.

On the other hand, overexpression of miR-223 in NRCs significantly increased glucose uptake, in similar magnitude to that observed in cells stimulated with insulin. *In vitro*, overexpression of miR-223 in cardiomyocytes increased total GLUT4 level and induced GLUT4 translocation from the cytoplasmic compartment to the cell membrane. *In vivo*, inhibition of miR-223 in the heart resulted in a significant decrease in GLUT4 expression (Lu et al., 2010). miR-141 may be another miRNA of importance in DCM, since it was found to be significantly increased in the diabetic heart and its overexpression decreased the content of the inner mitochondrial membrane phosphate transporter, solute carrier family 25 member 3 (Slc25a3) in the mouse atrial cardiomyocyte cell line HL-1 (Baseler et al., 2012), suggesting that altered miR-141 expression may influence cardiac mitochondrial function and ATP generation (Baseler et al., 2012).

Further extending the evidence for the role of miRNA in the pathophysiology DCM, miR-451 levels were found to be markedly increased in palmitate-stimulated NRCs and DIO mouse heart (model of pre-T2DM and obesity with elevated blood glucose and impaired glucose tolerance). It was found that Cab39 (Calcium-binding protein 39, a scaffold protein of LKB1, a kinase upstream of AMPK) was a direct target to miR-451 in the heart. Furthermore, miR-451 knockdown partly rescued lipotoxicity *in vitro*, and HFD-induced cardiac hypertrophy was ameliorated in miR-451 KO mice through the LKB1/AMPK pathway (Kuwabara et al., 2015). Moreover, activin A released from epicardial adipose tissue in T2DM, inhibits insulin action via the induction of miR-143 in cardiomyocytes. This miRNA inhibits the AKT pathway through down-regulation of the novel regulator of insulin action, ORP8 (Blumensatt et al., 2013). ORP8 binds 25-OH-cholesterol, and this regulates AKT signaling in apoptosis through the induction of AKT degradation via proteasome and/or inhibition of PI3K in macrophages (Jordan et al., 2011). Moreover, activation of AKT in response to insulin and the consequent phosphorylation of glycogen synthase kinase 3β (GSK3β) were diminished in HepG2 cells depleted of ORP8 (Jordan et al., 2011). Future studies should confirm these results in the heart to further clarify the role of miR-143 and ORP8 in DCM.

Regarding insulin, it has been shown that miR-29, which downregulates the pro-survival protein myeloid cell leukemia 1 (MCL-1) (Roggli et al., 2012), may be involved in the development of DCM. Arnold et al., reported that insulin inhibited the expression of miR-29a, b and c and increased the mRNA levels of MCL-1 in HL-1 cells. Additionally, they observed that dysregulation of the miR-29-MCL-1 axis as a consequence of low levels of insulin or inhibition of mTORC1 in Zucker Diabetic Fatty (ZDF) rats was associated with structural damage of the diabetic myocardium (Arnold et al., 2014). In addition, Marchand et al. have recently reported that miR-322 modulates insulin pathway, protecting the heart against the cardiac dysfunction observed in C57Bl/6N mice fed with HFD, showing that miR-322 overexpression reduced AKT phosphorylation induced by insulin. Thus, miR-322 regulates insulin signaling and might protect the heart against consequences of hyperinsulinemia (Marchand et al., 2016). Moreover, Costantino et al. showed that altered miRs profile induced by hyperglycaemia in the diabetic heart is not reverted by the restoration of normal glycaemia levels with insulin (Costantino et al., 2016). This study also showed through ingenuity pathway analysis that the miRs increased in the diabetic heart were involved in myocardial cell signaling pathways associated with apoptosis, fibrosis, hypertrophic growth, autophagy, oxidative stress, and HF and this may explain why the detrimental effects of DCM persist even after the restoration of normal glucose levels (Costantino et al., 2016). Considering all the evidence shown above, miRNAs clearly participate in the pathophysiological mechanisms involved in DCM and more studies are required to fully understand and control their diagnostic and therapeutic potential.

#### Exosomes

Exosomes are nanosized extracellular vesicles ranging from 50 to 90 nm in diameter. These nanovesicles are formed in multivesicular bodies, which after fusing with the plasma membrane; release their content to the extracellular medium (Thery et al., 2006). Exosomes can carry proteins, RNA or miRNA from one cell to another through the bloodstream, protecting its content from enzymatic degradation and thus, these extracellular vesicles may be mediating intercellular communications (Yellon and Davidson, 2014). Moreover, de Jong et al reported that cellular stress may induce variations in the protein and mRNA content of endothelial cell-derived exosomes (De Jong et al., 2012), which suggests that exosomes may represent the physiological state of the cell (Yellon and Davidson, 2014). Therefore, exosomes are a very attractive target to develop possible biomarkers for different diseases.

The potential role of exosomes in cardiovascular diseases is starting to be explored. In this context, it has been described that exosomes exert a cardioprotective effect in the myocardium against ischemia-reperfusion (Giricz et al., 2014; Vicencio et al., 2015). It has also been reported that exosomes may play a role in septic cardiomyopathy, cardiac hypertrophy, and peripartum cardiomyopathy (Ailawadi et al., 2015).

Regarding DCM and the importance of miRNAs in this disease, exosomes arise as potential biomarkers for diagnosis. However, much remains to be elucidated about these nanovesicles and their role in DCM. In this context, it has been suggested that most of the plasma miRNAs are contained in exosomes, however, it has also been reported that plasma miRNAs can be bound to complexes of proteins like argonaute or high density lipoproteins (Yellon and Davidson, 2014) and therefore, further studies are required to clarify this point.

In the context of understanding the mechanisms involved in diabetes-induced ischemic cardiac disease (Ailawadi et al., 2015), Wang et al showed that cardiomyocyte-derived exosomes isolated from Goto-Kakizaki (GK) diabetic rats inhibited the proliferation and migration of endothelial cells. Interestingly, exosomes isolated from Wistar rat's cardiomyocytes generated the opposite effect in the endothelial cells, promoting migration and proliferation. In addition, the effects of both GK and Wistar rat's exosomes on endothelial cells were reversed by the exosome inhibitor GW4869. Moreover, GK-exosomes contained higher levels of miR-320 and lower levels of miR-126 compared to Wistar rat's cardiomyocyte-derived exosomes (Wang et al., 2014). Therefore, these results suggest that reduced angiogenesis in the diabetic myocardium may be mediated by exosomes with anti-angiogenic effects released from cardiomyocytes to the endothelium. These findings also suggest a possible role for exosomes for the treatment of DCM ischemic complications.

It has also been recently reported that cardiomyocyte-derived exosomes can regulate glucose transport in endothelial cells (Garcia et al., 2016). This study showed that under glucose deprivation conditions, the production of exosomes from cardiomyocytes was increased and these nanovesicles contained glucose transporters and enzymes involved in glycolysis, which in turn lead to an increase in glucose uptake, glycolysis and the production of pyruvate in the endothelial cells (Garcia et al., 2016). Taken the current evidence together, exosomes have massive potential to give new insights that could help to understand the pathophysiology of DCM and they can not only serve as potential biomarkers, but also act as therapeutic targets or agents that could reverse the impaired insulin signaling observed in DCM.

#### Adenosine

The purine nucleoside adenosine is an important paracrine/autocrine signaling molecule that modulates a wide range of biological processes activating four subtypes of G protein-coupled adenosine receptors (ARs), A_1_, A_2*A*_, A_2*B*_, and A_3_ (Fredholm, 2014). The ARs play a key role in tissue homeostasis both in physiological and pathophysiological conditions, such as inflammation, ischemia, hypoxia and cancer, regulating tissue homeostasis (Antonioli et al., 2013).

Interestingly, new evidence has emerged suggesting a link between adenosine signaling and T2DM (Antonioli et al., 2015). In this context, it has been described that adenosine modulates insulin secretion (Johansson et al., 2007; Yang et al., 2012; Ohtani et al., 2013) and also controls the proliferation and regeneration of β pancreatic cells (Ohtani et al., 2013; Andersson, 2014). Moreover, several studies have proposed that adenosine regulates the insulin signaling in muscle (Faulhaber-Walter et al., 2011; Figler et al., 2011; Johnston-Cox et al., 2012), liver (Figler et al., 2011; Johnston-Cox et al., 2012), and adipose tissue (Faulhaber-Walter et al., 2011; Figler et al., 2011; Johnston-Cox et al., 2012; Csoka et al., 2014).

In the cardiovascular system, the expression of ARs have been reported in vascular smooth muscle cells (Zhao et al., 1997), human umbilical vein endothelial cells (Wyatt et al., 2002), coronary artery endothelial cells (Olanrewaju et al., 2000), cardiac fibroblasts and cardiomyocytes (Sassi et al., 2014). In STZ-treated diabetic rats, cardiomyocytes exhibited increased levels of A_1_ and A_3_, reduced levels of A_2A_ and unaltered levels of A_2B_ (Grden et al., 2005); however, cardiac fibroblasts showed increased levels of A_1_ and A_2A,_reduced levels of A_3_ but did not change the levels of A_2B_ (Grden et al., 2006). Thus, the evidence described above highlights the need to assess the functional role of ARs in DCM.

As we mentioned before, early-stage DCM is characterized by cardiomyocyte hypertrophy and myocardial fibrosis (Battiprolu et al., 2013; Go et al., 2014). In this context, Puhl et al. (2016) recently showed that A_1_ attenuates phenylephrine-mediated (α-1 adrenoceptor) cardiomyocyte hypertrophic and cardiac fibrosis, suggesting that A_1_ activation might be a novel therapeutic target to prevent cardiac remodeling (Puhl et al., 2016). In addition, cell-to-cell communication via cAMP/adenosine activating A_1_ and A_2A_/A_2B_ in cardiomyocytes and fibroblast, respectively, may be also relevant in preventing isoproterenol-mediated (βAR agonist) cardiomyocyte hypertrophy and cardiac fibrosis, suggesting an interesting paracrine cardiac action of adenosine (Sassi et al., 2014). In summary, whether ARs participates in the molecular mechanisms of DCM, or whether they might be promoting the DCM-related defective insulin signaling are important issues that need to be addressed.

## Concluding remarks and open questions

The complete pathophysiology of DCM and the role of insulin in it are still being elucidated. Further studies are certainly required to clarify many aspects of this cardiovascular disease. However, there has also been emerging information on different mechanisms that may help answer the variety of questions raised in the study of DCM. Despite increasing amount of evidence that highlights the role of ARs in the regulation of insulin signaling in several tissues such as muscle, liver and adipose tissue, there are many open questions to deeply understand their roles both in the healthy heart and DCM. Thus, could insulin signaling in DCM be improved by activation of ARs? In addition, miRNAs may play a key role for early diagnosis of DCM and evidence such as the altered miRNA landscape in the diabetic heart suggests that new strategies are required to reverse the detrimental effects of DCM that persists despite normalization of blood glucose levels. In this context, is it possible to reverse the cardiovascular complications of DCM by targeting the miRNAs associated with these deleterious effects? Also, exosomes may also serve as potential biomarkers of DCM. These nanovesicles are a mechanism of inter-cellular communication and therefore, there is much excitement about the possibility of harnessing their power as therapeutic agents. Could normal exosomes isolated from healthy patients reverse the phenotype of patients with DCM? Is it possible to exogenously control the release of beneficial exosomes? Answers to these questions will help to elucidate the complex mechanisms underlying the defective insulin signaling in DCM, opening new fields for basic and clinical studies.

## Author contributions

FW and SL contributed to conception and design the manuscript. JR, MP, VG, AD, HV, PC, and LG, drafted and critically revised the manuscript.

### Conflict of interest statement

The authors declare that the research was conducted in the absence of any commercial or financial relationships that could be construed as a potential conflict of interest.

## References

[B1] Abel E. D. (2004). Glucose transport in the heart. Front. Biosci. 9, 201–215. 10.2741/1216 14766360

[B2] Abel E. D. Graveleau C. Betuing S. Pham M. Reay P. A. Kandror V.. (2004). Regulation of insulin-responsive aminopeptidase expression and targeting in the insulin-responsive vesicle compartment of glucose transporter isoform 4-deficient cardiomyocytes. Mol. Endocrinol. 18, 2491–2501. 10.1210/me.2004-0175 15231875

[B3] Abel E. D. Kaulbach H. C. Tian R. Hopkins J. C. Duffy J. Doetschman T.. (1999). Cardiac hypertrophy with preserved contractile function after selective deletion of GLUT4 from the heart. J. Clin. Invest. 104, 1703–1714. 10.1172/JCI7605 10606624 PMC409881

[B4] Abel E. D. O'Shea K. M. Ramasamy R. (2012). Insulin resistance: metabolic mechanisms and consequences in the heart. Arterioscler. Thromb. Vasc. Biol. 32, 2068–2076. 10.1161/ATVBAHA.111.241984 22895668 PMC3646067

[B5] Adeghate E. Singh J. (2014). Structural changes in the myocardium during diabetes-induced cardiomyopathy. Heart Fail. Rev. 19, 15–23. 10.1007/s10741-013-9388-5 23467937

[B6] Ailawadi S. Wang X. Gu H. Fan G. C. (2015). Pathologic function and therapeutic potential of exosomes in cardiovascular disease. Biochim. Biophys. Acta 1852, 1–11. 10.1016/j.bbadis.2014.10.008 25463630 PMC4268281

[B7] Andersson O. (2014). Role of adenosine signalling and metabolism in beta-cell regeneration. Exp. Cell Res. 321, 3–10. 10.1016/j.yexcr.2013.11.019 24315942

[B8] Antonioli L. Blandizzi C. Csoka B. Pacher P. Hasko G. (2015). Adenosine signalling in diabetes mellitus–pathophysiology and therapeutic considerations. Nat. Rev. Endocrinol. 11, 228–241. 10.1038/nrendo.2015.10 25687993

[B9] Antonioli L. Blandizzi C. Pacher P. Hasko G. (2013). Immunity, inflammation and cancer: a leading role for adenosine. Nat. Rev. Cancer 13, 842–857. 10.1038/nrc3613 24226193

[B10] Arnlov J. Lind L. Zethelius B. Andren B. Hales C. N. Vessby B.. (2001). Several factors associated with the insulin resistance syndrome are predictors of left ventricular systolic dysfunction in a male population after 20 years of follow-up. Am. Heart J. 142, 720–724. 10.1067/mhj.2001.116957 11579365

[B11] Arnold N. Koppula P. R. Gul R. Luck C. Pulakat L. (2014). Regulation of cardiac expression of the diabetic marker microRNA miR-29. PLoS ONE 9:e103284. 10.1371/journal.pone.0103284 25062042 PMC4111545

[B12] Asrih M. Steffens S. (2013). Emerging role of epigenetics and miRNA in diabetic cardiomyopathy. Cardiovasc. Pathol. 22, 117–125. 10.1016/j.carpath.2012.07.004 22951386

[B13] Augustus A. S. Buchanan J. Park T. S. Hirata K. Noh H. L. Sun J.. (2006). Loss of lipoprotein lipase-derived fatty acids leads to increased cardiac glucose metabolism and heart dysfunction. J. Biol. Chem. 281, 8716–8723. 10.1074/jbc.M509890200 16410253

[B14] Banting F. G. Best C. H. Collip J. B. Campbell W. R. Fletcher A. A. (1922). Pancreatic extracts in the treatment of diabetes mellitus. Can. Med. Assoc. J. 12, 141–146. 20314060 PMC1524425

[B15] Baseler W. A. Thapa D. Jagannathan R. Dabkowski E. R. Croston T. L. Hollander J. M. (2012). miR-141 as a regulator of the mitochondrial phosphate carrier (Slc25a3) in the type 1 diabetic heart. Am. J. Physiol. Cell Physiol. 303, C1244–C1251. 10.1152/ajpcell.00137.2012 23034391 PMC3532490

[B16] Battiprolu P. K. Gillette T. G. Wang Z. V. Lavandero S. Hill J. A. (2010). Diabetic cardiomyopathy: mechanisms and therapeutic targets. Drug Discov. Today Dis. Mech. 7, e135–e143. 10.1016/j.ddmec.2010.08.001 21274425 PMC3026473

[B17] Battiprolu P. K. Hojayev B. Jiang N. Wang Z. V. Luo X. Iglewski M.. (2012). Metabolic stress-induced activation of FoxO1 triggers diabetic cardiomyopathy in mice. J. Clin. Invest. 122, 1109–1118. 10.1172/JCI60329 22326951 PMC3287230

[B18] Battiprolu P. K. Lopez-Crisosto C. Wang Z. V. Nemchenko A. Lavandero S. Hill J. A. (2013). Diabetic cardiomyopathy and metabolic remodeling of the heart. Life Sci. 92, 609–615. 10.1016/j.lfs.2012.10.011 23123443 PMC3593804

[B19] Belke D. D. Betuing S. Tuttle M. J. Graveleau C. Young M. E. Pham M.. (2002). Insulin signaling coordinately regulates cardiac size, metabolism, and contractile protein isoform expression. J. Clin. Invest. 109, 629–639. 10.1172/JCI0213946 11877471 PMC150890

[B20] Bell D. Mcdermott B. J. (2000). Troglitazone does not initiate hypertrophy but can sensitise cardiomyocytes to growth effects of serum. Eur. J. Pharmacol. 390, 237–244. 10.1016/S0014-2999(99)00932-2 10708729

[B21] Bertoni A. G. Hundley W. G. Massing M. W. Bonds D. E. Burke G. L. Goff D. C. Jr. (2004). Heart failure prevalence, incidence, and mortality in the elderly with diabetes. Diabetes Care 27, 699–703. 10.2337/diacare.27.3.699 14988288

[B22] Blumensatt M. Greulich S. Herzfeld De Wiza D. Mueller H. Maxhera B. Rabelink M. J.. (2013). Activin A impairs insulin action in cardiomyocytes via up-regulation of miR-143. Cardiovasc. Res. 100, 201–210. 10.1093/cvr/cvt173 23812417

[B23] Boudina S. Bugger H. Sena S. O'Neill B. T. Zaha V. G. Ilkun O.. (2009). Contribution of impaired myocardial insulin signaling to mitochondrial dysfunction and oxidative stress in the heart. Circulation 119, 1272–1283. 10.1161/CIRCULATIONAHA.108.792101 19237663 PMC2739097

[B24] Buchanan J. Mazumder P. K. Hu P. Chakrabarti G. Roberts M. W. Yun U. J.. (2005). Reduced cardiac efficiency and altered substrate metabolism precedes the onset of hyperglycemia and contractile dysfunction in two mouse models of insulin resistance and obesity. Endocrinology 146, 5341–5349. 10.1210/en.2005-0938 16141388

[B25] Bugger H. Abel E. D. (2014). Molecular mechanisms of diabetic cardiomyopathy. Diabetologia 57, 660–671. 10.1007/s00125-014-3171-6 24477973 PMC3969857

[B26] Bugger H. Boudina S. Hu X. X. Tuinei J. Zaha V. G. Theobald H. A.. (2008). Type 1 diabetic akita mouse hearts are insulin sensitive but manifest structurally abnormal mitochondria that remain coupled despite increased uncoupling protein 3. Diabetes 57, 2924–2932. 10.2337/db08-0079 18678617 PMC2570388

[B27] Bugger H. Riehle C. Jaishy B. Wende A. R. Tuinei J. Chen D.. (2012). Genetic loss of insulin receptors worsens cardiac efficiency in diabetes. J. Mol. Cell. Cardiol. 52, 1019–1026. 10.1016/j.yjmcc.2012.02.001 22342406 PMC3327790

[B28] Bushati N. Cohen S. M. (2007). microRNA functions. Annu. Rev. Cell Dev. Biol. 23, 175–205. 10.1146/annurev.cellbio.23.090506.123406 17506695

[B29] Cai L. Li W. Wang G. Guo L. Jiang Y. Kang Y. J. (2002). Hyperglycemia-induced apoptosis in mouse myocardium: mitochondrial cytochrome C-mediated caspase-3 activation pathway. Diabetes 51, 1938–1948. 10.2337/diabetes.51.6.1938 12031984

[B30] Chen S. Puthanveetil P. Feng B. Matkovich S. J. Dorn G. W. II. Chakrabarti S. (2014). Cardiac miR-133a overexpression prevents early cardiac fibrosis in diabetes. J. Cell. Mol. Med. 18, 415–421. 10.1111/jcmm.12218 24428157 PMC3955148

[B31] Contreras-Ferrat A. E. Toro B. Bravo R. Parra V. Vasquez C. Ibarra C.. (2010). An inositol 1,4,5-triphosphate (IP3)-IP3 receptor pathway is required for insulin-stimulated glucose transporter 4 translocation and glucose uptake in cardiomyocytes. Endocrinology 151, 4665–4677. 10.1210/en.2010-0116 20685879

[B32] Cook S. A. Varela-Carver A. Mongillo M. Kleinert C. Khan M. T. Leccisotti L.. (2010). Abnormal myocardial insulin signalling in type 2 diabetes and left-ventricular dysfunction. Eur. Heart J. 31, 100–111. 10.1093/eurheartj/ehp396 19797329 PMC2800920

[B33] Coort S. L. Bonen A. Van Der Vusse G. J. Glatz J. F. Luiken J. J. (2007). Cardiac substrate uptake and metabolism in obesity and type-2 diabetes: role of sarcolemmal substrate transporters. Mol. Cell. Biochem. 299, 5–18. 10.1007/s11010-005-9030-5 16988889 PMC1915649

[B34] Costantino S. Paneni F. Lüscher T. F. Cosentino F. (2016). MicroRNA profiling unveils hyperglycaemic memory in the diabetic heart. Eur Heart J. 37, 572–576. 10.1093/eurheartj/ehv599 26553540

[B35] Csoka B. Koscso B. Toro G. Kokai E. Virag L. Nemeth Z. H.. (2014). A2B adenosine receptors prevent insulin resistance by inhibiting adipose tissue inflammation via maintaining alternative macrophage activation. Diabetes 63, 850–866. 10.2337/db13-0573 24194503 PMC3931402

[B36] Cushman S. W. Wardzala L. J. (1980). Potential mechanism of insulin action on glucose transport in the isolated rat adipose cell. Apparent translocation of intracellular transport systems to the plasma membrane. J. Biol. Chem. 255, 4758–4762. 6989818

[B37] Das U. N. (2003). Insulin: an endogenous cardioprotector. Curr. Opin. Crit. Care 9, 375–383. 10.1097/00075198-200310000-00007 14508150

[B38] De Jong O. G. Verhaar M. C. Chen Y. Vader P. Gremmels H. Posthuma G.. (2012). Cellular stress conditions are reflected in the protein and RNA content of endothelial cell-derived exosomes. J. Extracell Vesicles 1:18396. 10.3402/jev.v1i0.18396 24009886 PMC3760650

[B39] Dhalla N. S. Takeda N. Rodriguez-Leyva D. Elimban V. (2014). Mechanisms of subcellular remodeling in heart failure due to diabetes. Heart Fail. Rev. 19, 87–99. 10.1007/s10741-013-9385-8 23436108

[B40] Diaz A. Humeres C. Gonzalez V. Gomez M. T. Montt N. Sanchez G.. (2015). Insulin/NFkappaB protects against ischemia-induced necrotic cardiomyocyte death. Biochem. Biophys. Res. Commun. 467, 451–457. 10.1016/j.bbrc.2015.09.171 26449460

[B41] Ebina Y. Edery M. Ellis L. Standring D. Beaudoin J. Roth R. A.. (1985). Expression of a functional human insulin receptor from a cloned cDNA in Chinese hamster ovary cells. Proc. Natl. Acad. Sci. U.S.A. 82, 8014–8018. 10.1073/pnas.82.23.8014 3906655 PMC391432

[B42] Falcao-Pires I. Leite-Moreira A. F. (2012). Diabetic cardiomyopathy: understanding the molecular and cellular basis to progress in diagnosis and treatment. Heart Fail. Rev. 17, 325–344. 10.1007/s10741-011-9257-z 21626163

[B43] Faulhaber-Walter R. Jou W. Mizel D. Li L. Zhang J. Kim S. M.. (2011). Impaired glucose tolerance in the absence of adenosine A1 receptor signaling. Diabetes 60, 2578–2587. 10.2337/db11-0058 21831968 PMC3178298

[B44] Figler R. A. Wang G. Srinivasan S. Jung D. Y. Zhang Z. Pankow J. S.. (2011). Links between insulin resistance, adenosine A2B receptors, and inflammatory markers in mice and humans. Diabetes 60, 669–679. 10.2337/db10-1070 21270276 PMC3028369

[B45] Finck B. N. Han X. Courtois M. Aimond F. Nerbonne J. M. Kovacs A.. (2003). A critical role for PPARalpha-mediated lipotoxicity in the pathogenesis of diabetic cardiomyopathy: modulation by dietary fat content. Proc. Natl. Acad. Sci. U.S.A. 100, 1226–1231. 10.1073/pnas.0336724100 12552126 PMC298755

[B46] Finck B. N. Lehman J. J. Leone T. C. Welch M. J. Bennett M. J. Kovacs A.. (2002). The cardiac phenotype induced by PPARalpha overexpression mimics that caused by diabetes mellitus. J. Clin. Invest. 109, 121–130. 10.1172/JCI0214080 11781357 PMC150824

[B47] Fiordaliso F. Li B. Latini R. Sonnenblick E. H. Anversa P. Leri A.. (2000). Myocyte death in streptozotocin-induced diabetes in rats in angiotensin II- dependent. Lab. Invest. 80, 513–527. 10.1038/labinvest.3780057 10780668

[B48] Fredholm B. B. (2014). Adenosine–a physiological or pathophysiological agent? J. Mol. Med. 92, 201–206. 10.1007/s00109-013-1101-6 24362516

[B49] Frustaci A. Kajstura J. Chimenti C. Jakoniuk I. Leri A. Maseri A.. (2000). Myocardial cell death in human diabetes. Circ. Res. 87, 1123–1132. 10.1161/01.RES.87.12.1123 11110769

[B50] Fu Q. Xu B. Liu Y. Parikh D. Li J. Li Y.. (2014). Insulin inhibits cardiac contractility by inducing a gi-biased beta2-adrenergic signaling in hearts. Diabetes 63, 2676–2689. 10.2337/db13-1763 24677713 PMC4113065

[B51] Garcia N. A. Moncayo-Arlandi J. Sepulveda P. Diez-Juan A. (2016). Cardiomyocyte exosomes regulate glycolytic flux in endothelium by direct transfer of GLUT transporters and glycolytic enzymes. Cardiovasc Res. 109, 397–408. 10.1093/cvr/cvv260 26609058

[B52] Giricz Z. Varga Z. V. Baranyai T. Sipos P. Paloczi K. Kittel A.. (2014). Cardioprotection by remote ischemic preconditioning of the rat heart is mediated by extracellular vesicles. J. Mol. Cell. Cardiol. 68, 75–78. 10.1016/j.yjmcc.2014.01.004 24440457

[B53] Glatz J. F. Bonen A. Ouwens D. M. Luiken J. J. (2006). Regulation of sarcolemmal transport of substrates in the healthy and diseased heart. Cardiovasc. Drugs Ther. 20, 471–476. 10.1007/s10557-006-0582-8 17119873

[B54] Go A. S. Mozaffarian D. Roger V. L. Benjamin E. J. Berry J. D. Blaha M. J.. (2014). Heart disease and stroke statistics–2014 update: a report from the American Heart Association. Circulation 129, e28–e292. 10.1161/01.cir.0000442015.53336.12 24352519 PMC5408159

[B55] Graveleau C. Zaha V. G. Mohajer A. Banerjee R. R. Dudley-Rucker N. Steppan C. M.. (2005). Mouse and human resistins impair glucose transport in primary mouse cardiomyocytes, and oligomerization is required for this biological action. J. Biol. Chem. 280, 31679–31685. 10.1074/jbc.M504008200 15983036

[B56] Grden M. Podgorska M. Kocbuch K. Szutowicz A. Pawelczyk T. (2006). Expression of adenosine receptors in cardiac fibroblasts as a function of insulin and glucose level. Arch. Biochem. Biophys. 455, 10–17. 10.1016/j.abb.2006.08.022 17011509

[B57] Grden M. Podgorska M. Szutowicz A. Pawelczyk T. (2005). Altered expression of adenosine receptors in heart of diabetic rat. J. Physiol. Pharmacol. 56, 587–597. 16391416

[B58] Horie T. Ono K. Nishi H. Iwanaga Y. Nagao K. Kinoshita M.. (2009). MicroRNA-133 regulates the expression of GLUT4 by targeting KLF15 and is involved in metabolic control in cardiac myocytes. Biochem. Biophys. Res. Commun. 389, 315–320. 10.1016/j.bbrc.2009.08.136 19720047

[B59] Ikeda H. Shiojima I. Ozasa Y. Yoshida M. Holzenberger M. Kahn C. R.. (2009). Interaction of myocardial insulin receptor and IGF receptor signaling in exercise-induced cardiac hypertrophy. J. Mol. Cell. Cardiol. 47, 664–675. 10.1016/j.yjmcc.2009.08.028 19744489 PMC4886750

[B60] Ingelsson E. Sundstrom J. Arnlov J. Zethelius B. Lind L. (2005). Insulin resistance and risk of congestive heart failure. JAMA 294, 334–341. 10.1001/jama.294.3.334 16030278

[B61] Isfort M. Stevens S. C. Schaffer S. Jong C. J. Wold L. E. (2014). Metabolic dysfunction in diabetic cardiomyopathy. Heart Fail. Rev. 19, 35–48. 10.1007/s10741-013-9377-8 23443849 PMC3683106

[B62] Jia G. DeMarco V. G. Sowers J. R. (2016). Insulin resistance and hyperinsulinaemia in diabetic cardiomyopathy. Nat. Rev. Endocrinol. 12, 144–153. 10.1038/nrendo.2015.216 26678809 PMC4753054

[B63] Johansson S. M. Salehi A. Sandstrom M. E. Westerblad H. Lundquist I. Carlsson P. O.. (2007). A1 receptor deficiency causes increased insulin and glucagon secretion in mice. Biochem. Pharmacol. 74, 1628–1635. 10.1016/j.bcp.2007.08.006 17869224

[B64] Johnston-Cox H. Koupenova M. Yang D. Corkey B. Gokce N. Farb M. G.. (2012). The A2b adenosine receptor modulates glucose homeostasis and obesity. PLoS ONE 7:e40584. 10.1371/journal.pone.0040584 22848385 PMC3405065

[B65] Jordan S. D. Kruger M. Willmes D. M. Redemann N. Wunderlich F. T. Bronneke H. S.. (2011). Obesity-induced overexpression of miRNA-143 inhibits insulin-stimulated AKT activation and impairs glucose metabolism. Nat. Cell Biol. 13, 434–446. 10.1038/ncb2211 21441927

[B66] Kahn C. R. Freychet P. Roth J. Neville D. M. Jr. (1974). Quantitative aspects of the insulin-receptor interaction in liver plasma membranes. J. Biol. Chem. 249, 2249–2257. 4362069

[B67] Kuwabara Y. Horie T. Baba O. Watanabe S. Nishiga M. Usami S.. (2015). MicroRNA-451 exacerbates lipotoxicity in cardiac myocytes and high-fat diet-induced cardiac hypertrophy in mice through suppression of the LKB1/AMPK pathway. Circ. Res. 116, 279–288. 10.1161/CIRCRESAHA.116.304707 25362209

[B68] Leto D. Saltiel A. R. (2012). Regulation of glucose transport by insulin: traffic control of GLUT4. Nat. Rev. Mol. Cell Biol. 13, 383–396. 10.1038/nrm3351 22617471

[B69] Levine R. Goldstein M. Klein S. Huddlestun B. (1949). The action of insulin on the distribution of galactose in eviscerated nephrectomized dogs. J. Biol. Chem. 179, 985–986. 18150030

[B70] Li Y. Wende A. R. Nunthakungwan O. Huang Y. Hu E. Jin H.. (2012). Cytosolic, but not mitochondrial, oxidative stress is a likely contributor to cardiac hypertrophy resulting from cardiac specific GLUT4 deletion in mice. FEBS J. 279, 599–611. 10.1111/j.1742-4658.2011.08450.x 22221582 PMC3267000

[B71] Lopez-Izquierdo A. Pereira R. O. Wende A. R. Punske B. B. Abel E. D. Tristani-Firouzi M. (2014). The absence of insulin signaling in the heart induces changes in potassium channel expression and ventricular repolarization. Am. J. Physiol. Heart Circ. Physiol. 306, H747–H754. 10.1152/ajpheart.00849.2013 24375641 PMC3949065

[B72] Lu H. Buchan R. J. Cook S. A. (2010). MicroRNA-223 regulates Glut4 expression and cardiomyocyte glucose metabolism. Cardiovasc. Res. 86, 410–420. 10.1093/cvr/cvq010 20080987

[B73] Luiken J. J. Koonen D. P. Willems J. Zorzano A. Becker C. Fischer Y.. (2002). Insulin stimulates long-chain fatty acid utilization by rat cardiac myocytes through cellular redistribution of FAT/CD36. Diabetes 51, 3113–3119. 10.2337/diabetes.51.10.3113 12351456

[B74] Maisch B. Alter P. Pankuweit S. (2011). Diabetic cardiomyopathy–fact or fiction? Herz 36, 102–115. 10.1007/s00059-011-3429-4 21424347

[B75] Marchand A. Atassi F. Mougenot N. Clergue M. Codoni V. Berthuin J.. (2016). miR-322 regulates insulin signaling pathway and protects against metabolic syndrome-induced cardiac dysfunction in mice. Biochim. Biophys. Acta. 1862, 611–621. 10.1016/j.bbadis.2016.01.010 26775030

[B76] Mazumder P. K. O'Neill B. T. Roberts M. W. Buchanan J. Yun U. J. Cooksey R. C.. (2004). Impaired cardiac efficiency and increased fatty acid oxidation in insulin-resistant ob/ob mouse hearts. Diabetes 53, 2366–2374. 10.2337/diabetes.53.9.2366 15331547

[B77] Mbanya J. C. Sobngwi E. Mbanya D. S. Ngu K. B. (2001). Left ventricular mass and systolic function in African diabetic patients: association with microalbuminuria. Diabetes Metab. 27, 378–382. 11431604

[B78] McQueen A. P. Zhang D. Hu P. Swenson L. Yang Y. Zaha V. G.. (2005). Contractile dysfunction in hypertrophied hearts with deficient insulin receptor signaling: possible role of reduced capillary density. J. Mol. Cell. Cardiol. 39, 882–892. 10.1016/j.yjmcc.2005.07.017 16216265

[B79] Murarka S. Movahed M. R. (2010). Diabetic cardiomyopathy. J. Card. Fail. 16, 971–979. 10.1016/j.cardfail.2010.07.249 21111987

[B80] Ni Y. G. Berenji K. Wang N. Oh M. Sachan N. Dey A.. (2006). Foxo transcription factors blunt cardiac hypertrophy by inhibiting calcineurin signaling. Circulation 114, 1159–1168. 10.1161/CIRCULATIONAHA.106.637124 16952979 PMC4118290

[B81] Ni Y. G. Wang N. Cao D. J. Sachan N. Morris D. J. Gerard R. D.. (2007). FoxO transcription factors activate Akt and attenuate insulin signaling in heart by inhibiting protein phosphatases. Proc. Natl. Acad. Sci. U.S.A. 104, 20517–20522. 10.1073/pnas.0610290104 18077353 PMC2154463

[B82] Ohtani M. Oka T. Ohura K. (2013). Possible involvement of A(2)A and A(3) receptors in modulation of insulin secretion and beta-cell survival in mouse pancreatic islets. Gen. Comp. Endocrinol. 187, 86–94. 10.1016/j.ygcen.2013.02.011 23453966

[B83] Olanrewaju H. A. Qin W. Feoktistov I. Scemama J. L. Mustafa S. J. (2000). Adenosine A(2A) and A(2B) receptors in cultured human and porcine coronary artery endothelial cells. Am. J. Physiol. Heart Circ. Physiol. 279, H650–H656. 10924064 10.1152/ajpheart.2000.279.2.H650

[B84] Olson E. N. Schneider M. D. (2003). Sizing up the heart: development redux in disease. Genes Dev. 17, 1937–1956. 10.1101/gad.1110103 12893779

[B85] O'Neill B. T. Kim J. Wende A. R. Theobald H. A. Tuinei J. Buchanan J.. (2007). A conserved role for phosphatidylinositol 3-kinase but not Akt signaling in mitochondrial adaptations that accompany physiological cardiac hypertrophy. Cell Metab. 6, 294–306. 10.1016/j.cmet.2007.09.001 17908558 PMC2084219

[B86] Ozasa N. Furukawa Y. Morimoto T. Tadamura E. Kita T. Kimura T. (2008). Relation among left ventricular mass, insulin resistance, and hemodynamic parameters in type 2 diabetes. Hypertens. Res. 31, 425–432. 10.1291/hypres.31.425 18497461

[B87] Pasquinelli A. E. (2012). MicroRNAs and their targets: recognition, regulation and an emerging reciprocal relationship. Nat. Rev. Genet. 13, 271–282. 10.1038/nrg3162 22411466

[B88] Pasumarthi K. B. Field L. J. (2002). Cardiomyocyte cell cycle regulation. Circ. Res. 90, 1044–1054. 10.1161/01.RES.0000020201.44772.67 12039793

[B89] Poulsen M. K. Henriksen J. E. Dahl J. Johansen A. Gerke O. Vach W.. (2010). Left ventricular diastolic function in type 2 diabetes mellitus: prevalence and association with myocardial and vascular disease. Circ. Cardiovasc. Imaging 3, 24–31. 10.1161/CIRCIMAGING.109.855510 19846730

[B90] Pritchard C. C. Cheng H. H. Tewari M. (2012). MicroRNA profiling: approaches and considerations. Nat. Rev. Genet. 13, 358–369. 10.1038/nrg3198 22510765 PMC4517822

[B91] Puhl S. L. Kazakov A. Müller A. Fries P. Wagner D. R. Böhm M.. (2016). A1 receptor activation attenuates cardiac hypertrophy and fibrosis in response to alpha1-adrenergic stimulation in vivo. Br. J. Pharmacol. 173, 88–102. 10.1111/bph.13339 26406609 PMC4813379

[B92] Punske B. B. Rossi S. Ershler P. Rasmussen I. Abel E. D. (2004). Optical mapping of propagation changes induced by elevated extracellular potassium ion concentration in genetically altered mouse hearts. J. Electrocardiol. 37(suppl.), 128–134. 10.1016/j.jelectrocard.2004.08.037 15534822

[B93] Qi Y. Zhu Q. Zhang K. Thomas C. Wu Y. Kumar R.. (2015). Activation of Foxo1 by insulin resistance promotes cardiac dysfunction and beta-myosin heavy chain gene expression. Circ. Heart Fail. 8, 198–208. 10.1161/CIRCHEARTFAILURE.114.001457 25477432 PMC9241109

[B94] Richey P. A. Brown S. P. (1998). Pathological versus physiological left ventricular hypertrophy: a review. J. Sports Sci. 16, 129–141. 10.1080/026404198366849 9531002

[B95] Riehle C. Wende A. R. Sena S. Pires K. M. Pereira R. O. Zhu Y.. (2013). Insulin receptor substrate signaling suppresses neonatal autophagy in the heart. J. Clin. Invest. 123, 5319–5333. 10.1172/JCI71171 24177427 PMC3859408

[B96] Riehle C. Wende A. R. Zhu Y. Oliveira K. J. Pereira R. O. Jaishy B. P.. (2014). Insulin receptor substrates are essential for the bioenergetic and hypertrophic response of the heart to exercise training. Mol. Cell. Biol. 34, 3450–3460. 10.1128/MCB.00426-14 25002528 PMC4135616

[B97] Roggli E. Gattesco S. Caille D. Briet C. Boitard C. Meda P.. (2012). Changes in microRNA expression contribute to pancreatic beta-cell dysfunction in prediabetic NOD mice. Diabetes 61, 1742–1751. 10.2337/db11-1086 22537941 PMC3379668

[B98] Rubler S. Dlugash J. Yuceoglu Y. Z. Kumral T. Branwood A. W. Grishman A. (1972). New type of cardiomyopathy associated with diabetic glomerulosclerosis. Am. J. Cardiol. 30, 595–602. 10.1016/0002-9149(72)90595-4 4263660

[B99] Sassi Y. Ahles A. Truong D. J. Baqi Y. Lee S. Y. Husse B.. (2014). Cardiac myocyte-secreted cAMP exerts paracrine action via adenosine receptor activation. J. Clin. Invest. 124, 5385–5397. 10.1172/JCI74349 25401477 PMC4297204

[B100] Schips T. G. Wietelmann A. Hohn K. Schimanski S. Walther P. Braun T.. (2011). FoxO3 induces reversible cardiac atrophy and autophagy in a transgenic mouse model. Cardiovasc. Res. 91, 587–597. 10.1093/cvr/cvr144 21628326

[B101] Sena S. Hu P. Zhang D. Wang X. Wayment B. Olsen C.. (2009). Impaired insulin signaling accelerates cardiac mitochondrial dysfunction after myocardial infarction. J. Mol. Cell. Cardiol. 46, 910–918. 10.1016/j.yjmcc.2009.02.014 19249310 PMC2683200

[B102] Sena S. Rasmussen I. R. Wende A. R. McQueen A. P. Theobald H. A. Wilde N.. (2007). Cardiac hypertrophy caused by peroxisome proliferator- activated receptor-gamma agonist treatment occurs independently of changes in myocardial insulin signaling. Endocrinology 148, 6047–6053. 10.1210/en.2006-1559 17823261

[B103] Sengupta A. Molkentin J. D. Yutzey K. E. (2009). FoxO transcription factors promote autophagy in cardiomyocytes. J. Biol. Chem. 284, 28319–28331. 10.1074/jbc.M109.024406 19696026 PMC2788882

[B104] Shaw J. E. Sicree R. A. Zimmet P. Z. (2010). Global estimates of the prevalence of diabetes for 2010 and 2030. Diabetes Res. Clin. Pract. 87, 4–14. 10.1016/j.diabres.2009.10.007 19896746

[B105] Shen W. H. Chen Z. Shi S. Chen H. Zhu W. Penner A.. (2008). Cardiac restricted overexpression of kinase-dead mammalian target of rapamycin (mTOR) mutant impairs the mTOR-mediated signaling and cardiac function. J. Biol. Chem. 283, 13842–13849. 10.1074/jbc.M801510200 18326485 PMC2376248

[B106] Shimizu I. Minamino T. Toko H. Okada S. Ikeda H. Yasuda N.. (2010). Excessive cardiac insulin signaling exacerbates systolic dysfunction induced by pressure overload in rodents. J. Clin. Invest. 120, 1506–1514. 10.1172/JCI40096 20407209 PMC2860916

[B107] Shioi T. Kang P. M. Douglas P. S. Hampe J. Yballe C. M. Lawitts J.. (2000). The conserved phosphoinositide 3-kinase pathway determines heart size in mice. EMBO J. 19, 2537–2548. 10.1093/emboj/19.11.2537 10835352 PMC212739

[B108] Shiojima I. Yefremashvili M. Luo Z. Kureishi Y. Takahashi A. Tao J.. (2002). Akt signaling mediates postnatal heart growth in response to insulin and nutritional status. J. Biol. Chem. 277, 37670–37677. 10.1074/jbc.M204572200 12163490

[B109] Skurk C. Izumiya Y. Maatz H. Razeghi P. Shiojima I. Sandri M.. (2005). The FOXO3a transcription factor regulates cardiac myocyte size downstream of AKT signaling. J. Biol. Chem. 280, 20814–20823. 10.1074/jbc.M500528200 15781459 PMC3632436

[B110] Sohn K. Wende A. R. Abel E. D. Moreno A. P. Sachse F. B. Punske B. B. (2013). Absence of glucose transporter 4 diminishes electrical activity of mouse hearts during hypoxia. Exp. Physiol. 98, 746–757. 10.1113/expphysiol.2012.070235 23180812 PMC6599691

[B111] Son N. H. Yu S. Tuinei J. Arai K. Hamai H. Homma S.. (2010). PPARgamma-induced cardiolipotoxicity in mice is ameliorated by PPARalpha deficiency despite increases in fatty acid oxidation. J. Clin. Invest. 120, 3443–3454. 10.1172/JCI40905 20852389 PMC2947216

[B112] Sonoyama K. Greenstein A. Price A. Khavandi K. Heagerty T. (2007). Vascular remodeling: implications for small artery function and target organ damage. Ther. Adv. Cardiovasc. Dis. 1, 129–137. 10.1177/1753944707086358 19124402

[B113] Suzuki K. Kono T. (1980). Evidence that insulin causes translocation of glucose transport activity to the plasma membrane from an intracellular storage site. Proc. Natl. Acad. Sci. U.S.A. 77, 2542–2545. 10.1073/pnas.77.5.2542 6771756 PMC349437

[B114] Thery C. Amigorena S. Raposo G. Clayton A. (2006). Isolation and characterization of exosomes from cell culture supernatants and biological fluids. Curr. Protoc. Cell Biol. Chapter 3, Unit 3.22. 10.1002/0471143030.cb0322s30 18228490

[B115] Tian R. Abel E. D. (2001). Responses of GLUT4-deficient hearts to ischemia underscore the importance of glycolysis. Circulation 103, 2961–2966. 10.1161/01.CIR.103.24.2961 11413087

[B116] Troncoso R. Ibarra C. Vicencio J. M. Jaimovich E. Lavandero S. (2014). New insights into IGF-1 signaling in the heart. Trends Endocrinol. Metab. 25, 128–137. 10.1016/j.tem.2013.12.002 24380833

[B117] Ullrich A. Bell J. R. Chen E. Y. Herrera R. Petruzzelli L. M. Dull T. J.. (1985). Human insulin receptor and its relationship to the tyrosine kinase family of oncogenes. Nature 313, 756–761. 10.1038/313756a0 2983222

[B118] Vered A. Battler A. Segal P. Liberman D. Yerushalmi Y. Berezin M.. (1984). Exercise-induced left ventricular dysfunction in young men with asymptomatic diabetes mellitus (diabetic cardiomyopathy). Am. J. Cardiol. 54, 633–637. 10.1016/0002-9149(84)90263-7 6475785

[B119] Vicencio J. M. Yellon D. M. Sivaraman V. Das D. Boi-Doku C. Arjun S.. (2015). Plasma exosomes protect the myocardium from ischemia-reperfusion injury. J. Am. Coll. Cardiol. 65, 1525–1536. 10.1016/j.jacc.2015.02.026 25881934

[B120] Voulgari C. Papadogiannis D. Tentolouris N. (2010). Diabetic cardiomyopathy: from the pathophysiology of the cardiac myocytes to current diagnosis and management strategies. Vasc. Health Risk Manag. 6, 883–903. 10.2147/VHRM.S11681 21057575 PMC2964943

[B121] Wang X. Huang W. Liu G. Cai W. Millard R. W. Wang Y.. (2014). Cardiomyocytes mediate anti-angiogenesis in type 2 diabetic rats through the exosomal transfer of miR-320 into endothelial cells. J. Mol. Cell. Cardiol. 74, 139–150. 10.1016/j.yjmcc.2014.05.001 24825548 PMC4120246

[B122] Wright J. J. Kim J. Buchanan J. Boudina S. Sena S. Bakirtzi K.. (2009). Mechanisms for increased myocardial fatty acid utilization following short-term high-fat feeding. Cardiovasc. Res. 82, 351–360. 10.1093/cvr/cvp017 19147655 PMC2675931

[B123] Wyatt A. W. Steinert J. R. Wheeler-Jones C. P. Morgan A. J. Sugden D. Pearson J. D.. (2002). Early activation of the p42/p44MAPK pathway mediates adenosine-induced nitric oxide production in human endothelial cells: a novel calcium-insensitive mechanism. FASEB J. 16, 1584–1594. 10.1096/fj.01-0125com 12374781

[B124] Yang G. K. Fredholm B. B. Kieffer T. J. Kwok Y. N. (2012). Improved blood glucose disposal and altered insulin secretion patterns in adenosine A(1) receptor knockout mice. Am. J. Physiol. Endocrinol. Metab. 303, E180–E190. 10.1152/ajpendo.00050.2012 22550063

[B125] Yellon D. M. Davidson S. M. (2014). Exosomes: nanoparticles involved in cardioprotection? Circ. Res. 114, 325–332. 10.1161/circresaha.113.300636 24436428

[B126] Zhao S. M. Wang Y. L. Guo C. Y. Chen J. L. Wu Y. Q. (2014). Progressive decay of Ca^2+^ homeostasis in the development of diabetic cardiomyopathy. Cardiovasc. Diabetol. 13:75. 10.1186/1475-2840-13-75 24712865 PMC3991902

[B127] Zhao Z. Francis C. E. Ravid K. (1997). An A3-subtype adenosine receptor is highly expressed in rat vascular smooth muscle cells: its role in attenuating adenosine-induced increase in cAMP. Microvasc. Res. 54, 243–252. 10.1006/mvre.1997.2044 9441895

[B128] Zheng D. Ma J. Yu Y. Li M. Ni R. Wang G.. (2015). Silencing of miR-195 reduces diabetic cardiomyopathy in C57BL/6 mice. Diabetologia 58, 1949–1958. 10.1007/s00125-015-3622-8 25994075 PMC4499474

[B129] Zhu Y. Pereira R. O. O'Neill B. T. Riehle C. Ilkun O. Wende A. R.. (2013a). Cardiac PI3K-Akt impairs insulin-stimulated glucose uptake independent of mTORC1 and GLUT4 translocation. Mol. Endocrinol. 27, 172–184. 10.1210/me.2012-1210 23204326 PMC3545208

[B130] Zhu Y. Pires K. M. Whitehead K. J. Olsen C. D. Wayment B. Zhang Y. C.. (2013b). Mechanistic target of rapamycin (Mtor) is essential for murine embryonic heart development and growth. PLoS ONE 8:e54221. 10.1371/journal.pone.0054221 23342106 PMC3544830

[B131] Zhu Y. Soto J. Anderson B. Riehle C. Zhang Y. C. Wende A. R.. (2013c). Regulation of fatty acid metabolism by mTOR in adult murine hearts occurs independently of changes in PGC-1alpha. Am. J. Physiol. Heart Circ. Physiol. 305, H41–H51. 10.1152/ajpheart.00877.2012 23624629 PMC3727103

